# Laparoscopic sacrohysteropexy versus vaginal hysterectomy and apical suspension: 7-year follow-up of a randomized controlled trial

**DOI:** 10.1007/s00192-021-04932-6

**Published:** 2021-08-23

**Authors:** Matthew L. Izett-Kay, Philip Rahmanou, Rufus J. Cartwright, Natalia Price, Simon R. Jackson

**Affiliations:** 1 Department of Urogynaecology, Women’s Centre, The John Radcliffe Hospital, Oxford University Hospitals, Headington, Oxford, OX3 9FR UK; 2 UCL EGA Institute for Women’s Health, University College London, Medical School Building, 74 Huntley Street, London, WC1E 6AU UK; 3 Department of Urogynaecology, Gloucestershire Hospitals NHS Foundation Trust, Gloucestershire, Gloucester, GL13NN UK

**Keywords:** Pelvic organ prolapse, Laparoscopy, Vaginal hysterectomy, Laparoscopic sacrohysteropexy, Mesh

## Abstract

**Introduction and hypothesis:**

Laparoscopic mesh sacrohysteropexy offers a uterine-sparing alternative to vaginal hysterectomy with apical suspension, although randomised comparative data are lacking. This study was aimed at comparing the long-term efficacy of laparoscopic mesh sacrohysteropexy and vaginal hysterectomy with apical suspension for the treatment of uterine prolapse.

**Methods:**

A randomised controlled trial comparing laparoscopic mesh sacrohysteropexy and vaginal hysterectomy with apical suspension for the treatment of uterine prolapse was performed, with a minimum follow-up of 7 years. The primary outcome was reoperation for apical prolapse. Secondary outcomes included patient-reported mesh complications, Pelvic Organ Prolapse Quantification, Patient Global Impression of Improvement in prolapse symptoms and the International Consultation on Incontinence Questionnaire Vaginal Symptoms, Female Lower Urinary Tract Symptoms (ICIQ-FLUTS) and PISQ-12 questionnaires.

**Results:**

A total of 101 women were randomised and 62 women attended for follow-up at a mean of 100 months postoperatively (range 84–119 months). None reported a mesh-associated complication. The risk of reoperation for apical prolapse was 17.2% following vaginal hysterectomy (VH) and 6.1% following laparoscopic mesh sacrohysteropexy (LSH; relative risk 0.34, 95% CI 0.07–1.68, *p* = 0.17). Laparoscopic sacrohysteropexy was associated with a statistically significantly higher apical suspension (POP-Q point C −5 vs −4.25, *p* = 0.02) and longer total vaginal length (9 cm vs 6 cm, *p* < 0.001). There was no difference in the change in ICIQ-VS scores between the two groups (ICIQ-VS change −22 vs −25, *p* = 0.59).

**Conclusion:**

Laparoscopic sacrohysteropexy and vaginal hysterectomy with apical suspension have comparable reoperation rates and subjective outcomes. Potential advantages of laparoscopic sacrohysteropexy include a lower risk of apical reoperation, greater apical support and increased total vaginal length.

**Supplementary Information:**

The online version contains supplementary material available at 10.1007/s00192-021-04932-6

## Introduction

A woman’s lifetime risk of surgery for pelvic organ prolapse (POP) is between 11% and 19% [1]. Rates of surgical intervention are predicted to increase [2] as populations age. The preferred surgical approach for uterine prolapse amongst many urogynaecologists remains vaginal hysterectomy (VH) with apical suspension [3], recognising the association between the more common anterior wall prolapse and apical descent [3, 4]. However, high-quality randomised controlled trial (RCT) data suggest that this approach might be associated with a high surgical failure rate of up to 35% at 2 years [5]. The risk of subsequent reoperation for posthysterectomy vault prolapse is between 4.6% and 18% [6, 7]. Most women with uterine prolapse would prefer to avoid hysterectomy if an equally effective alternative were available [8]. It is therefore unsurprising that in many countries, including the USA and the UK, rates of uterine-preserving prolapse procedures are increasing [9, 10].

Laparoscopic mesh sacrohysteropexy (LSH) is one such uterine-preserving alternative that appears to be safe [11], yet comparative data supporting its use remain sparse. A systematic review found only two randomised controlled trials comparing abdominal hysteropexy with VH with apical suspension, and both reported only short-term outcomes [12]. A multicentre trial in the Netherlands (*N* = 82) compared open sacrohysteropexy with VH, and found comparable rates of subjective prolapse symptom scores but higher reoperation and medical consultation for prolapse after hysteropexy compared with VH at 12 months [13]. An earlier 12-month follow-up of the RCT reported here compared LSH with VH and apical suspension; although the level of apical suspension, vaginal length, hospital stay, return to activities and blood loss all favoured LSH, there was no significant difference in risk of reoperation for apical prolapse [14].

More recently, the Vault or Uterine prolapse Evaluation (VUE) study, a large multicentre RCT, attempted to address this lack of evidence, comparing VH with either abdominal or vaginal hysteropexy [15]. The trial did not meet its recruitment target and only 23% of women in the uterine preservation arm received an abdominal hysteropexy [15], meaning that the results may not be generalisable to LSH. The majority received a vaginal uterine suspension procedure, yet this technique is known to be associated with a high failure rate [16].

Given the trends in prolapse surgery, ageing populations, changing patient desires and the high failure rate associated with VH, the merits of abdominal hysteropexy deserve further scrutiny. In this report, we analysed the long-term outcomes at 7 years of our earlier RCT, which compared VH plus apical suspension with LSH [14].

## Materials and methods

We conducted a non-blinded, single-centre, multi-surgeon RCT comparing mesh-augmented LSH with VH plus apical suspension for the treatment of uterine prolapse, undertaken at the John Radcliffe Hospital, Oxford, UK, from May 2009 to September 2012. This study was originally approved by the National Research Ethics Committee (reference number: 09/H0606/28). In order to undertake the 7-year follow-up that was not part of the original protocol a substantial amendment was made, approved along with Health Research Agency approval on 8 January 2019 by the South Central Oxford C REC. Our inclusion and exclusion criteria, as well as recruitment and randomisation, were described in our earlier report [14]. Following REC approval of the amendment to allow for further follow-up, study participants were contacted by telephone and invited for a study visit, and those who attended completed further written consent. Telephone verbal consent was obtained for those who were happy to undertake a telephone history and send a postal questionnaire. Following two attempts at telephone contact all study participants were contacted by post with an information leaflet, patient-reported outcome measures (PROMs) and study-specific questionnaire to capture primary and secondary outcomes. The return of these questionnaires was taken as implied consent. The case notes of all women randomised in the original study were additionally reviewed by a single researcher (MI).

We have previously described the surgical technique carried out in our unit for LSH [17]. This involves the use of a bifurcated polypropylene mesh, either PRO-Lite™ (Atrium Medical Corporation, Hudson, NH, USA) or Prolene™ mesh (Ethicon, Somerville, NJ, USA), wrapped around the cervix through broad ligament windows and secured anteriorly with non-absorbable sutures (Ethibond Excel™; Ethicon), that is then secured to the sacral promontory with a helical fastener (Protack™; United States Surgical, Tyco Healthcare, Norwalk, CT, USA). For VH, a modified McCall’s culdoplasty was performed, with the uterosacral ligaments reattached to the vaginal vault with absorbable sutures (Vicryl 1; Ethicon, Somerville, NJ, USA). For those participants with procidentia, additional vault support was obtained with a sacrospinous fixation. This utilised absorbable sutures (PDS II 0; Ethicon), mirroring common clinical practice and in keeping with UK national recommendations [18]. Both LSH and VH were combined with anterior and/or posterior repair on the basis of intraoperative assessment and judgement at the time of surgery by the operating surgeon. Generally, anterior or posterior wall prolapse above the hymen was left, unless explicitly planned preoperatively following the patient’s wishes.

The primary objective of this study was to compare the efficacy of mesh-augmented LSH and VH between the two groups by determining the risk of reoperation for apical prolapse. Therefore, the primary outcome measure was subsequent reoperation for apical prolapse within the study period. Secondary outcomes included reoperation for any POP, POP status according to Pelvic Organ Prolapse Quantification (POP-Q), diagnosis of a mesh-associated complication, and subjective pelvic floor outcomes from a number of International Continence Society (ICS) grade A PROMs, including the International Consultation on Incontinence Questionnaire Vaginal Symptoms (ICIQ-VS), the International Consultation on Incontinence Questionnaire-Female Lower Urinary Tract Symptoms (ICIQ-FLUTS) and the Pelvic Organ Prolapse/Urinary Incontinence Sexual Questionnaire IUGA revised (PISQ-IR).

The original trial was run as a pilot with no formal power calculation. Retrospectively, we can estimate that the sample size provided approximately 80% power at the 12-month follow-up for a 25% difference in the primary outcome. Data for the whole study population were subject to descriptive statistics. For the primary outcome of a dichotomous variable of either having had reoperation for apical prolapse or not, a Chi-squared test was performed. This was used for all other dichotomous variables. Parametric data were subject to Student’s *t* test and non-parametric data such as PROM scores were subject to a Mann–Whitney *U* test, all requiring a significance level set at *p* < 0.05. These were all analysed on an intention-to-treat basis. Kaplan–Meier survival analyses were also undertaken using the primary outcome as a failure variable. Statistical analysis was carried out using Stata/SE 15® (StataCorp, College Station, TX, USA).

## Results

**Fig. 1:**
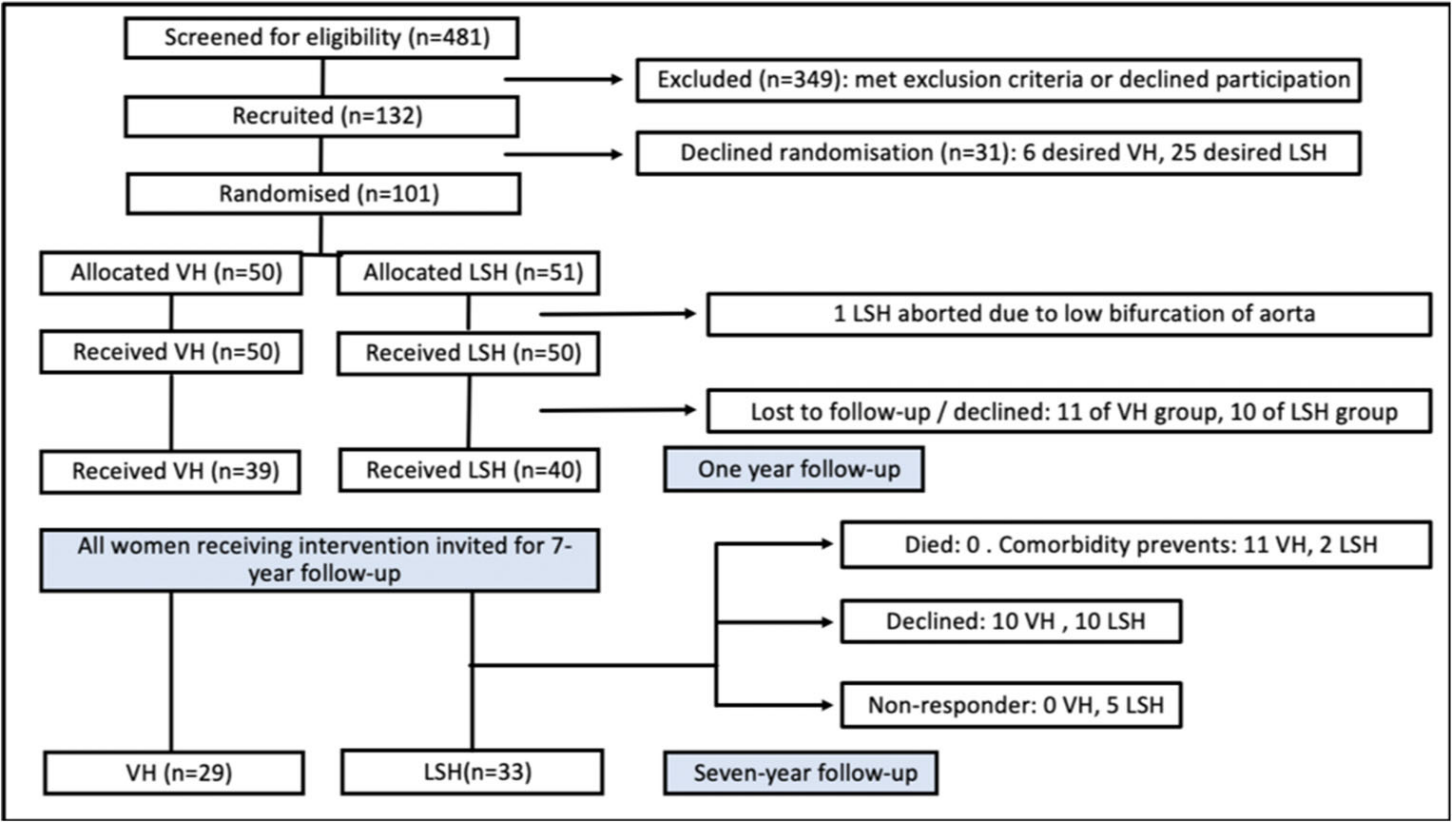
Consolidated Standards of Reporting Trials diagram. *LSH* laparoscopic mesh sacrohysteropexy, *VH* vaginal hysterectomy

Over the recruitment period, 481 women were invited to participate and 132 were recruited; however, 31 of these women later withdrew owing to a desire for a specific surgical procedure. A further patient randomised to LSH had an intraoperative conversion to VH because of a low bifurcation of the aorta precluding safe access to the sacral promontory. For the 1-year follow-up, data were available for 79 participants [14]. For the 7-year follow-up, 62 women (62%) provided outcome data, with a mean length of follow-up of 100 months (range 84–119 months) (see Consolidated Standards of Reporting Trials diagram, Fig. 1). A summary of the demographics comparing the two intervention arms for those with 7-year follow-up is shown in Table 1, with the VH group having a larger average POP-Q genital hiatus (GH) (4 vs 5, *p* = 0.002). Testing for non-responder bias, the only significant difference was slightly lower pre-operative ICIQ-VS scores in those who attended long-term follow-up (37.1 vs 31.8, *p* = 0.04), shown in Table 2.

**Table 1:** Baseline demographic data at initial recruitment for those with 7-year follow-up

Baseline characteristics	LSH	VH	*p* value
	*n* = 33	*n* = 29	
Age, years	64.13 ± 7.08	66.22 ± 6.15	0.20
BMI, kg/m_2_	25.86 (19–35)	26.99 (19–35)	0.24
Parity	2 (1–5)	2 (1–4)	0.42
Preoperative ICIQ-VS	31.58 ± 12.61	32 ± 12.15	0.92
Preoperative ICIQ-VS-SM	28.63 ± 15.16	25.27 ± 20.52	0.49
Preoperative ICIQ-VS-QOL	6.58 ± 2.54	7.74 ± 2.30	0.08
Length follow-up, months (range)	99 (84–119)	95 (86–114)	0.39*
POP-Q parameters			
Ba	1 ± 2.20	1 ± 2.51	0.61
C	1 ± 2.65	1 ± 3.45	0.21
Bp	0 ± 2.60	0 ± 2.45	0.51
GH	5 ± 0.72	5 ± 0.86	0.20
TVL	8 ± 0.80	8 ± 1.24	0.95

Continuous data are listed as mean ± SD (Mann–Whitney *U* test), except for BMI and parity, which are median and interquartile range or *n* (%)

Mann–Whitney test was used for significance

*BMI* body mass index, *GH* genital hiatus, *ICIQ-VS* International Consultation on Incontinence Questionnaire Vaginal Symptoms, *LSH* laparoscopic mesh sacrohysteropexy, *POP-Q* Pelvic Organ Prolapse Quantification, *QOL* quality of life, *SM* sexual matters, *TVL* total vaginal length, *VH* vaginal hysterectomy

*Student’s *t* test

**Table 2:** Comparison of demographic data between those who attended 7-year follow-up and those who did not

	No 7-year follow-up	7-year follow-up	*p* value
	*n* = 39	*n* = 62	
Age, years	63.95 ± 9.81	65.11 ± 6.69	0.88
BMI, kg/m^2^	27.24 (20–37)	26.39 (19–36)	0.29
Parity	2 (1–6)	2 (1–5)	0.95
Preoperative ICIQ-VS	37.11 ± 10.12	31.77 ± 12.31	0.04
Preoperative ICIQ-VS-SM	29.42 ± 17.02	27.52 ± 16.89	0.67
Preoperative ICIQ0VS-QOL	7.81 ± 1.51	7.05 ± 2.49	0.19
POP-Q parameters			
Ba	1 ± 2.42	1 ± 2.32	0.62
C	2 ± 2.55	2 ± 3.02	0.79
Bp	0 ± 2.36	0 ± 2.53	0.36
GH	5 ± 0.75	5 ± 0.84	0.85
TVL	8 ± 0.65	8 ± 1.01	0.80

Continuous data are listed as mean ± SD (Mann–Whitney *U* test), except for BMI and parity, which are median and interquartile range or *n* (%)

*BMI* body mass index, *GH* genital hiatus, *ICIQ-VS* International Consultation on Incontinence Questionnaire Vaginal Symptoms, *POP-Q* Pelvic Organ Prolapse Quantification, *QOL* quality of life, *SM* sexual matters, *TVL* total vaginal length

Mann–Whitney test was used for significance

*Student’s *t* test

**Fig. 2:**
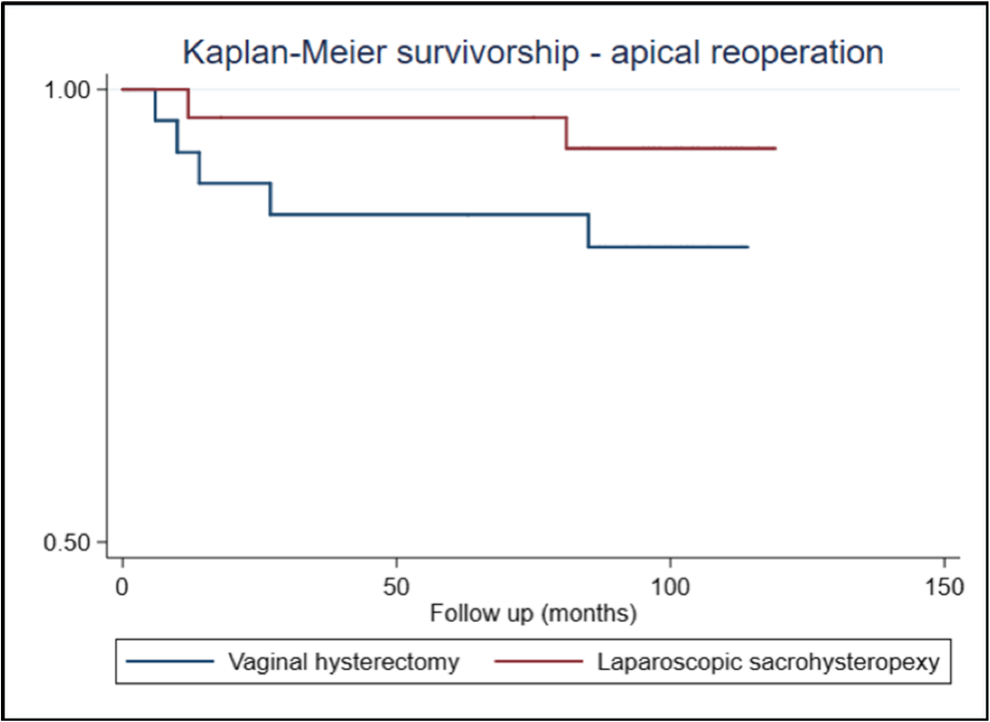
Kaplan–Meier survivorship using primary outcome as the failure variable

For the primary outcome of reoperation for apical prolapse, 6.1% of participants underwent such a procedure following LSH compared with 17.2% following VH; however, the difference was not statistically significant (relative risk [RR] 0.34, 95% CI 0.07–1.68, *p* = 0.17). A Kaplan–Meier survival analysis graph comparing the two groups based on the primary outcome is shown in Fig. 2. The nature of conservative and surgical interventions undertaken for all forms of recurrent POP are shown in Table 3. These results were not different when comparing a composite outcome of apical reoperation or apical anatomical failure. Reoperation rates from a case notes review of those with and without 7-year follow-up are shown in Table 4, with no significant difference between groups.

**Table 3:** Re-treatment for POP at 7 years

Follow-up data	LSH (*n* = 33)	VH (*n* = 29)	*p* value
Subsequent treatment for POP, %	9 (27.3)	7 (24.1)	0.78
Recurrent apical POP (reoperated apex or C≥ −1), %	5 (15.1)	7 (24.1)	0.37
Subsequent surgery for POP, %	6 (18.2)	6 (20.7)	0.80
Apical			
LSH, %	2 (6.1)	–	0.17
SCP, %	–	5 (17.2)	
Colporrhaphy			
Anterior colporrhaphy, %	2 (6.1)	–	–
Anterior and posterior colporrhaphy, %	2 (6.1)	–	–
Posterior colporrhaphy, %	–	1 (3.4)	–
PFMT, %	2 (6.1)	1 (3.4)	0.63
Pessary, %	1 (3)	–	0.34

Categorical data are listed as *n* (%) with Chi-squared testing

*LSH* laparoscopic mesh sacrohysteropexy, *PFMT* pelvic floor muscle training, *POP* pelvic organ prolapse, *SCP* sacrocolpopexy, *VH* vaginal hysterectomy

**Table 4:** Reoperation rates from a case notes review for all women enrolled in the study

Follow-up data	LSH (*n* = 51)	VH (*n* = 50)	*p* value
Subsequent treatment for POP, %	14 (27.5)	11 (22)	0.53
Subsequent surgery for POP, %	9 (17.6)	10 (20)	0.76
Apical			
LSH, %	3 (5.8)		0.13
SCP, %		9 (1.8)	
VH, %	1 (2)		
Colporrhaphy, %	5 (9.8)	1 (2)	0.09
PFMT, %	2 (3.9)	–	0.15
Pessary, %	3 (0.6)	1 (2)	0.31

Categorical data are listed as *n* (%) with Chi-squared testing

*LSH* laparoscopic mesh sacrohysteropexy, *PFMT* pelvic floor muscle training, *POP* pelvic organ prolapse, *SCP* sacrocolpopexy, *VH* vaginal hysterectomy

### Objective outcomes

There were no reported cases of mesh removal surgery, mesh erosion or chronic pain attributed to the mesh in the LSH group. The POP-Q parameters are shown in Table 5, with statistically significantly higher apical suspension following sacrohysteropexy (POP-Q point C −5 vs −4.25, *p* = 0.02) and longer TVL (9 cm vs 6 cm, *p* = <0.001). There was no difference in the percentage of patients with POP-Q point C≤ −2 between the two groups (84.6% after VH, 81.2% after LSH, *p* = 0.73), or in the percentage of patients with POP-Q point C≤ 0 (92.3% after VH, 90.1% after LSH, *p* = 0.82).

**Table 5:** Subjective outcome data and Pelvic Organ Prolapse Quantification (POP-Q)

	LSH (*n* = 33)	VH (*n* = 29)	*p* value
Change in ICIQ-VS, mean	−22.39 ± 13.06	−24.91 ± 14.05	0.59
Postoperative ICIQ-VS-SM, mean	7.42 ± 13.15	1.28 ± 3.40	0.42
Postoperative ICIQ-VS-QOL, mean	1.42 ± 1.98	1.03 ± 1.72	0.43
Positive response to ICIQ-VS Q5, %	15 (45.5)	9 (31)	0.24*
POP-Q			
Ba (cm)	−1 ± 1.69	−0.5 ± 1.70	0.99
C (cm)	−5 ± 2.58	−4.25 ± 2.92	0.02
Bp (cm)	−2 ± 1.68	−2 ± 0.54	–
GH (cm)	3 ± 0.88	3 ± 0.88	0.97
TVL (cm)	9 ± 3.0	6 ± 1.20	<0.01
PGI-I (1–2), %	25 (75.8)	25 (86.2)	0.30*
ICIQ-FLUTS, mean	9.42 ± 5.95	9.53 ± 5.97	0.97
ICIQ-FLUTS_F, mean	3.39 ± 1.97	3.86± 2.08	0.46
ICIQ=FLUTS_V, mean	1.70 ± 1.94	1.76 ± 1.57	0.53
ICIQ-FLUTS_I, mean	4.33 ± 4.26	3.89 ± 3.42	0.88
PISQ-IR, mean	16.67 ± 3.67	13.75 ± 5.72	0.28

All values are mean ± standard deviation with Mann–Whitney used to test significance with the exception of POP-Q with median values

*FLUTS* Female Lower Urinary Tract Symptoms, *F* filling, *GH* genital hiatus, *I* incontinence, *ICIQ-VS* International Consultation on Incontinence Questionnaire Vaginal Symptoms, *PGI-I* Patient Global Impression of Improvement, *PISQ-IR* Pelvic Organ Prolapse/Urinary Incontinence Sexual Questionnaire IUGA revised, *QOL* quality of life, *SM* sexual matters, *TVL* total vaginal length, *V* voiding

*Dichotomous outcome of either positive or negative response to ICIQ-VS question 5 and yes or no to PGI-I 1 and 2, where Chi-squared test was used

### Subjective outcomes

Differences in the mean change in ICIQ-VS scores between those undergoing LSH and those undergoing VH were not statistically significant (Table 5). Data presented also illustrate that there was no difference between the two groups with respect to the other PROM scores, including the 7-year postoperative ICIQ-VS SM and QOL subscales, the composite ICIQ-FLUTS score as well as the filling, voiding and incontinence subscales, and the PISQ-12. Likewise, when analysing the likelihood of “awareness of a lump or bulge coming down in the vagina” (Q5 of the ICIQ-VS), a symptom determined by consensus to be an accepted marker of symptomatic prolapse, there was no difference between the two groups (RR 0.68, 95% CI 0.35 to 1.32, *p* = 0.24) [19]. The likelihood of patients reporting their prolapse symptoms as “very much better” or “much better” was 86% after VH and 76% after LSH (*p* = 0.29).

## Discussion

Our data show a non-significant, lower rate of apical reoperation following LSH compared with VH in the long term. The study was underpowered for this outcome. After 7 years, the objective success rate based on apical reoperation was 83% after VH and 94% after LSH. The POP-Q parameters TVL and point C suggest that there might be anatomical advantages to LSH, relevant as longer vaginal length and higher apical support are both features of normal vaginal anatomy and therefore surrogates for optimal surgical correction. Utilising a range of validated and internationally recommended PROMS, we found no difference between the treatment arms, with the exception of composite ICIQ-VS scores, which are confounded by different rates of concomitant surgery and significant differences in responder and non-responder ICIQ-VS scores. Our previously reported 1-year data showed no major intraoperative complications in either group. Total operating times were shorter in those having VH by a mean difference of 11.4 min (*p* < 0.001). However, estimated blood loss (EBL), length of hospital stay, pain scores and time returning to normal activity all favour LSH.

Considering our findings within the context of previously reported randomised and observational cohort studies is difficult owing to the heterogeneity of reported outcome measures and current lack of consensus for core outcome measures when studying pelvic floor disorders [20]. Unsurprisingly, our findings at 7 years appear to mirror previous observational data reported by our group, which also found no difference in apical reoperation rates [14, 21, 22]. The only comparable RCT utilised an open approach and did not report reoperation rates; however, subsequent reporting of their long-term data at a mean of 94 months in the form of a conference abstract found no significant difference in rates of reoperation (26% following hysteropexy, 14% following VH, *p* = 0.28) [13, 23].

More recently, one arm of the multicentre VUE study reported a 12-month follow-up for women randomised to either VH or hysteropexy [15]. Only 69 women (24.7%) underwent an abdominal approach to hysteropexy, 66 of whom (23.6%) had LSH. Data for the individual procedures were not provided and therefore direct comparison with our own study is difficult. The study found no significant difference between reoperation rates for prolapse (3.3% following VH, 6.1% following uterine preservation (OR 2.01 CI 0.81 to 4.95, *p* = 0.120). Although these rates are lower than that in our study, comparison of absolute rates at 12 months with our longer-term outcomes is not possible. A large prospective study that compared LSH and VH also reported no difference in reoperation rates between the two interventions [24]. However, this was not an RCT and small numbers at follow-up and significant differences between the baseline characteristics of the two cohorts make meaningful comparison difficult.

If our primary outcome is used as the definition of objective failure, the long-term cure rate of 94% following LSH is similar to the findings from the two largest, medium-term cohort studies of the procedure, which reported rates of 95% at 48 months and 98% at 3 months respectively [25, 26]. However, both defined failure based on anatomical prolapse; one using POP-Q point C of ≤ 0 as a cut-off for objective success and another using POP-Q point C of ≤ −2, an evidence-based discriminator for symptomatic prolapse [27]. Secondary analyses of our data show comparable success rates using these same anatomical cut-offs (POP-Q point C of ≤ −2, 81.3% following LSH and 84.6% following VH; POP-Q point C of ≤ 0, 93% following LSH and 92% following VH), with no significant difference between the two intervention arms. Another large case series reported an 80% cure rate based on POP-Q point C of≤ 0 in 138 women at 12 months, against which our 93% cure rate using this outcome measure for the LSH cohort compares favourably [28]. A large non-randomised parallel cohort study did find that POP-Q parameters Ba, Bp and GH favoured LSH, yet there was no difference in POP-Q point C. Our anatomical outcomes mirror our earlier data, with POP-Q point C and TVL favouring LSH [14].

Our reoperation rates for any form of recurrent prolapse, 18.2% following LSH and 20.7% following VH, are higher than those found in large data sets. The latest Cochrane review reported a reoperation rate for POP following vaginal surgery for apical prolapse of 9.3%, albeit with a heterogenous group of procedures within the meta-analysis rendering a comparison of limited value [29]. A recent large population study of 7,247 patients at a median of 5 years reported reoperation rates of 30%, 7% and 11% after sacrospinous hysteropexy, Manchester repair and VH respectively [16]. The largest series reporting on posthysterectomy vault prolapse would suggest a reoperation rate of between 6% and 11.6% [30, 31]. This may reflect the longer-term follow-up, as well as a clinical approach that avoids operation on mild prolapse during the primary procedure. Concurrent anterior and posterior compartment repair for prolapse above the hymenal ring is generally avoided, as we endeavour to avoid excessive vaginal surgery that could lead to dyspareunia, and evidence suggests that such prolapse might be less likely to be symptomatic and might be considered normal [27].

Given the impact of prolapse on QOL, it could be argued that PROMS may represent the most important measure when determining the impact of surgical interventions [32]. The RCT comparing open abdominal sacrohysteropexy with VH focused predominantly on patient-reported data, utilising QOL questionnaires they found that lower urinary tract symptoms, mobility and postoperative pain favoured VH [13]. There were similar findings between the groups on clinical assessment of prolapse and they concluded that there were no significant advantages to sacrohysteropexy. The VUE study corroborates the findings of our own data, reporting no significant difference in their primary outcome, prolapse symptoms based on POP-SS at 12 months, between the two groups, or in prolapse-associated QOL [15]. Most patients in the two groups had an ongoing feeling of something coming down (30.7% and 28.9% respectively), rates that compare favourably with our own longer-term symptom status results.

A parallel cohort study comparing LSH and VH also reported no difference in reoperation rates between the two interventions, or in POP-Q point C or in subjective outcomes [24]. They did find that POP-Q parameters Ba, Bp and GH favoured LSH, yet a lack of randomisation, small numbers at follow-up and significant differences between the baseline characteristics of the two cohorts make meaningful comparison difficult. The Cochrane meta-analysis reported “awareness of prolapse” based on validated questionnaires and provided a risk of 13.7% at 2 years following vaginal surgery; however, this group included a number of procedures, some of which utilised mesh, making comparison with our figures of 45.5% and 31% (after LSH and VH respectively) difficult [29].

### Implications

Our study findings should not lead to an alteration in clinical practice owing to the lack of statistical power and the resulting inability to detect a statistically significant difference in the primary outcome measures. However, absolute reoperation rates and types of reoperation may be informative for patient decision making, i.e. in keeping with earlier studies and observational data that there may be some advantages to LSH as outlined. For women seeking to avoid the risk of major reoperation for prolapse or wanting to maintain normal vaginal anatomy, LSH may offer advantages. However, our data illustrate no significant difference in reoperation rate or functional outcomes, regardless of the choice of intervention.

This study illustrates the feasibility of long-term randomised studies of surgical interventions for prolapse. Although underpowered, with a significant loss to follow-up, more robustly designed and larger prospective studies will allow for the much-needed direct comparison of surgical procedures, particularly with respect to the role of mesh augmentation and abdominal approaches for those troubled by POP. The trend towards lower apical reoperation risks in our data suggest that this might be an important focus for such work. Because single studies rarely change clinical practice, our data, as well as those from the VUE studies and other prospective work, are likely to form part of further meta-analysis.

### Strengths and limitations

The principle strengths of our study include the use of randomisation with comparable baseline demographics in both intervention arms, adherence to a pre-stated primary outcome and long-term follow-up. Our chosen primary outcome is important, as apical reoperation in the form of sacrocolpopexy and sacrospinous fixation have relatively higher morbidity than simple colporrhaphy [33]. Weight is added by the use of patient-reported data, gathered through the use of validated measures. This will allow for future inclusion in meta-analyses, which is important, as our study was not adequately powered for the primary outcome measure. Shortcomings include baseline differences in preoperative ICIQ-VS scores between those who did and those who did not attend follow-up and concurrent vaginal surgery rates between the two intervention arms, as well as a large loss to follow-up, which is common in long-term randomised studies of surgical interventions. Finally, follow-up observations were undertaken by a researcher (MI) not blinded to the patient’s primary intervention, potentially introducing observer bias.

## Conclusion

Our study illustrates that LSH and VH are both effective and safe interventions for uterine prolapse. With reasonably low rates of reoperation and good symptomatic resolution across the range of pelvic floor disorders, women can be confident in both options. Given a trend towards differences in apical reoperation rates and advantages with respect to TVL and POP-Q point C, some may opt to choose LSH over VH; however, this study does not definitively support such an advantage. Larger trials are needed for more precise estimates to inform practice, but these data will contribute to any future meta-analyses.

## Supplementary Information

ESM 1 (PDF 105 kb)

ESM 2 (EML 7 kb)
