# Supplementary material for: Laparoscopic sacrohysteropexy versus vaginal hysterectomy and apical suspension: 7-year follow-up of a randomized controlled trial
Source: Int Urogynecol J. 2021 Aug 23;33(7):1957–65. doi: 10.1007/s00192-021-04932-6 (PMC9270299; doi:10.1007/s00192-021-04932-6)
Supplement: Supplementary file 1 — (PDF 105 kb) [file 192_2021_4932_MOESM1_ESM.pdf]

Health Research Authority

South Central - Oxford C Research Ethics Committee

Level 3, Block B
Whitefriars Building
Lewins Mead
Bristol
BS1 2NT
Tel: 020 7104 8049

<u>Please note: This is the favourable opinion of the REC only and does not allow</u> the amendment to be implemented at NHS sites in England until the outcome of the HRA assessment has been confirmed.

08 January 2019

Mr Simon Robert Jackson
Consultant Gynaecologist
Oxford Radcliffe Hospitals Trust
Women's Centre, John Radcliffe Hosp
Headley Way, Headington, Oxford
OX3 9DU

Dear Mr Jackson

**Study title:**	**Laparoscopic hysteropexy versus vaginal hysterectomy for treatment of uterovaginal prolapse: a prospective randomised study.**
**REC reference:**	**09/H0606/28**
**EudraCT number:**	**N/A**
**Amendment number:**	**1**
**Amendment date:**	**18 December 2018**
**IRAS project ID:**	**11721**

The Committee considered an amendment to include a longer term six year follow up. The study will therefore be extended until 01/09/2019 to allow for all study participants to attend for follow up.

The above amendment was reviewed by the Sub-Committee in correspondence.

Ethical opinion

The members of the Committee taking part in the review gave a favourable ethical opinion of the amendment on the basis described in the notice of amendment form and supporting documentation.

Approved documents

The documents reviewed and approved at the meeting were:

Document	*Version*	*Date*
Covering letter on headed paper [REC Cover Letter 14.9 MI]		18 December 2018
Letters of invitation to participant [Cover Letter 17.9.2018]	1	17 September 2018
Notice of Substantial Amendment (non-CTIMP) [AmendmentForm_ReadyForSubmission]	1	18 December 2018
Participant information sheet (PIS) [PIS 15.11]	1	14 September 2018
Research protocol or project proposal [28.11.2018 V2 VHvLSH]	2	14 September 2018
Validated questionnaire [Gen PISQ12]		
Validated questionnaire [ICIQ-FLUTS]		
Validated questionnaire [ICIQ-VS]		
Validated questionnaire [PGI-I]		

Membership of the Committee

The members of the Committee who took part in the review are listed on the attached sheet.

Working with NHS Care Organisations

Sponsors should ensure that they notify the R&D office for the relevant NHS care organisation of this amendment in line with the terms detailed in the categorisation email issued by the lead nation for the study.

Statement of compliance

The Committee is constituted in accordance with the Governance Arrangements for Research Ethics Committees and complies fully with the Standard Operating Procedures for Research Ethics Committees in the UK.

We are pleased to welcome researchers and R & D staff at our Research Ethics Committee members' training days – see details at http://www.hra.nhs.uk/hra-training/

09/H0606/28: Please quote this number on all correspondence

Yours sincerely
PP

Mr David Carpenter
Chair

E-mail: nrescommittee.southcentral-oxfordc@nhs.net

Enclosures: *List of names and professions of members who took part in the review*

Copy to:

South Central - Oxford C Research Ethics Committee

Attendance at Sub-Committee of the REC meeting in correspondence.

Committee Members:

Name	*Profession*	*Present*	*Notes*
Mr David Carpenter	Retired Social Scientist	Yes	Meeting Chair
Mrs Rebekah Howe	Farmer	Yes	

Also in attendance:

Name	*Position (or reason for attending)*
Miss Alison Doherty	REC Assistant
Miss Charlotte Ferris	REC Manager
